# Successfully Treated Calcific Uremic Arteriolopathy: Two Cases of a High Anion Gap Metabolic Acidosis with Intravenous Sodium Thiosulfate

**DOI:** 10.1155/2014/765134

**Published:** 2014-11-19

**Authors:** Joshua L. Rein, Kana N. Miyata, Kobena A. Dadzie, Steven J. Gruber, Roxana Sulica, James F. Winchester

**Affiliations:** ^1^Department of Medicine, Mount Sinai Beth Israel, 350 E 17th Street, 20th Floor Baird Hall, New York, NY 10003, USA; ^2^Division of Nephrology and Hypertension, Mount Sinai Beth Israel, 350 E 17th Street, 18th Floor Baird Hall, New York, NY 10003, USA; ^3^Department of Pulmonary, Critical Care & Sleep Medicine, Mount Sinai Beth Israel, 7 Dazian Building, 1st Avenue at 16th Street, New York, NY 10003, USA

## Abstract

Calcific uremic arteriolopathy (CUA) is a rare and potentially fatal disorder of calcification involving subcutaneous small vessels and fat in patients with renal insufficiency. We describe the successful use of intravenous sodium thiosulfate (STS) for the treatment of CUA in two patients. The first case was complicated by the development of a severe anion gap metabolic acidosis, which was accompanied by a seizure. Both patients had complete wound healing within five months. Although STS should be considered in the treatment of CUA, little is known about pharmacokinetics and additional studies are required to determine dosing strategies to minimize severe potential side effects.

## 1. Introduction

Calcific uremic arteriolopathy (CUA), also known as calciphylaxis, is a rare but potentially fatal disorder associated with renal insufficiency. The condition is characterized by arteriolar calcification, thrombus formation, and distal ischemia and necrosis of the skin and subcutaneous tissue. Mortality is near 50% and is often due to ulcer-related sepsis. Risk factors include hypercalcemia, hyperphosphatemia, elevated calcium (Ca) and inorganic phosphorus (Pi) product, hyperparathyroidism, use of calcium-based phosphate binders, use of Vitamin D analogs, warfarin use or Vitamin K deficiency, female gender, peritoneal dialysis (PD), and Caucasian race [1].

Sodium thiosulfate (STS) has been traditionally used as an antidote for cyanide poisoning, cisplatin toxicity, and nephrocalcinosis. In 2004, Cicone et al. were the first to describe the successful use of STS for the treatment of calciphylaxis [2]. Since then, STS has been shown in numerous case reports to improve wound healing and diminish pain within days of initiation. We describe the successful use of intravenous STS for the treatment of CUA in two patients.

## 2. Case Presentation

### 2.1. Case 1

A 49-year-old Caucasian female presented with multiple tender lower extremity lesions that progressed over seven months. Past medical history was significant for end stage renal disease (ESRD) on continuous ambulatory PD and severe idiopathic pulmonary arterial hypertension (IPAH) on epoprostenol and warfarin. A skin biopsy revealed metastatic calcification consistent with calcinosis cutis.

**Figure 1:**
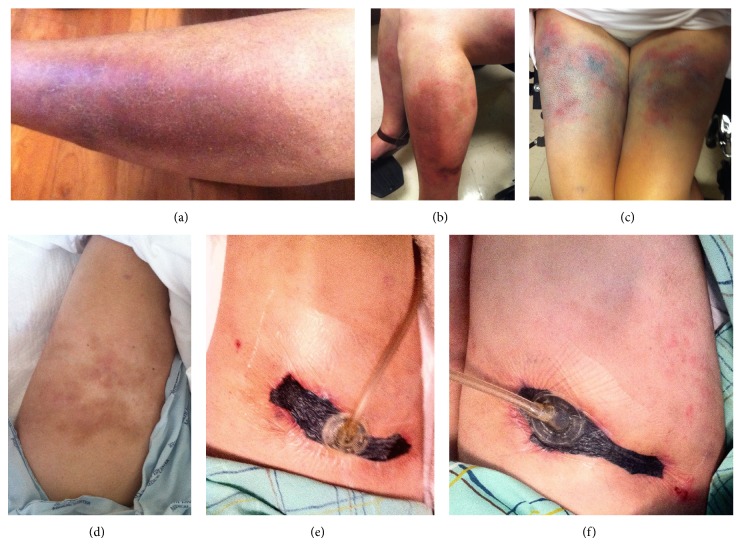
(a) Arm before treatment, (b) leg before treatment, (c) thighs before treatment, (d) left thigh 4 weeks after treatment, (e) right thigh with negative pressure dressing after 4 weeks of treatment, and (f) right thigh with negative pressure dressing after 8 weeks of treatment.

Upon presentation, physical exam showed severely painful, focal areas of necrosis surrounded by erythematous patches involving the proximal and distal upper and lower extremities (Figure 1). Laboratory studies were significant for Na 127 mmol/L, Cl 87 mmol/L, HCO_3_ 25 mmol/L, BUN 16.8 mmol/L, Ca 1.9 mmol/L, Pi 1.8 mmol/L, Ca × Pi 3.4, hemoglobin 95 g/L, albumin 3.3 *μ*mol/L, and intact PTH 33.00 pmol/L. STS 25 g was administered daily intravenously (IV) and warfarin was discontinued. Shortly after the start of STS, there was rapid improvement in her pain as the wounds began to heal.

The patient continued STS IV after discharge and developed a severe anion gap metabolic acidosis (AGMA) (Na 139 mmol/L, Cl 81 mmol/L, HCO_3_ 8 mmol/L, anion gap 50 mmol/L), which induced respiratory distress and a seizure. She was admitted to the intensive care unit where STS was held and sodium bicarbonate was administered. Serum bicarbonate level was normalized prior to restarting STS 12.5 g every other day. With the exception of nausea, the reduced dosing regimen was tolerated well with continued ulcer healing (Figure 1). At discharge, laboratory studies were notable for Na 135 mmol/L, Cl 91 mmol/L, HCO_3_ 31 mmol/L, Ca 1.9 mmol/L, Pi 1.7 mmol/L, Ca × Pi 3.2, and albumin 3.2 *μ*mol/L. After discharge, STS was continued at 12.5 g weekly for 4 months with complete healing. Sodium bicarbonate was continued at 1300 mg PO TID, titrated down, and eventually discontinued to maintain a serum bicarbonate level between 24 and 30 mmol/L. The patient died one year later from complications of IPAH.

### 2.2. Case 2

A 51-year-old morbidly obese Caucasian male presented with a one month history of multiple painful ulcerations on his calves. Past medical history is significant for stage 5 chronic kidney disease secondary to NSAID nephropathy and glomerulonephritis requiring 6 years of hemodialysis (HD) that the patient self-discontinued 5 years prior to this admission. The patient was noncompliant with his home medications. Skin biopsy revealed endoluminal thrombogenic calcification involving small subcutaneous blood vessels and interstitial calcification of adipocytes.

**Figure 2:**
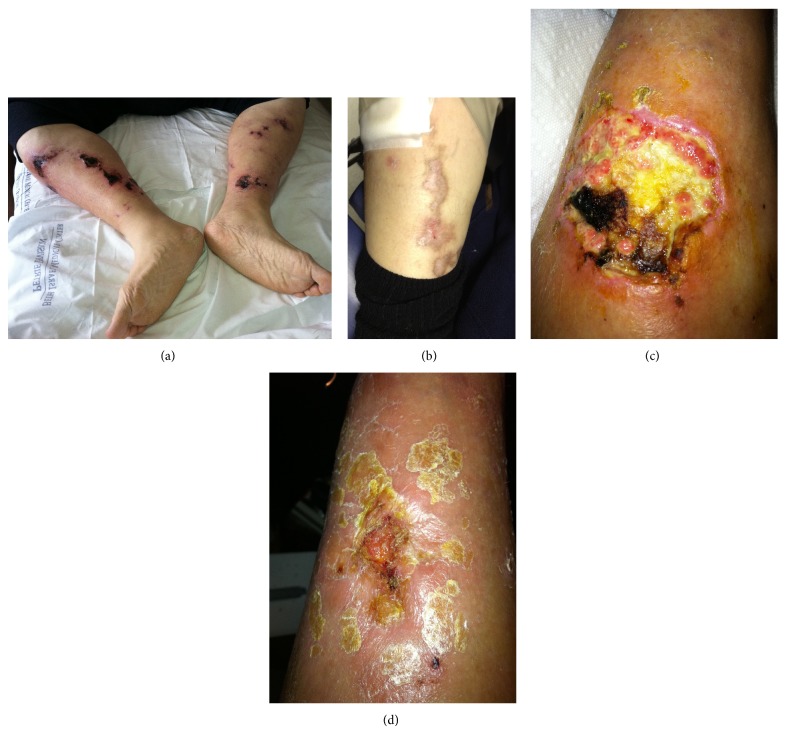
(a) Legs before treatment, (b) leg lesion after 12 weeks of treatment, (c) skin ulceration of forearm at beginning of treatment, and (d) skin ulceration of forearm after 8 weeks of treatment.

On presentation, physical exam revealed a large ulceration of left forearm and multiple dry and tender necrotic ulcers of the distal lower extremities (Figure 2). Laboratory studies were significant for Na 138 mmol/L, Cl 105 mmol/L, HCO_3_ 16 mmol/L, BUN 33.6 mmol/L, Ca 2.3 mmol/L, Pi 3.1 mmol/L, Ca × Pi 7.1, hemoglobin 78 g/L, albumin 4.9 *μ*mol/L, and intact PTH 375.61 pmol/L. The patient progressed to ESRD and was started on HD. Serum bicarbonate level was normalized prior to beginning STS. 25 g IV was dosed after dialysis three times weekly with the development of an AGMA and tolerated without significant side effects (Na 137 mmol/L, Cl 92 mmol/L, HCO_3_ 18 mmol/L, anion gap 27 mmol/L). Skin ulcerations showed significant healing and laboratory studies at discharge demonstrated Na 136 mmol/L, Cl 91 mmol/L, HCO_3_ 21 mmol/L, Ca 1.8 mmol/L, Pi 2.2 mmol/L, Ca × Pi 4.0, and intact PTH 173.1 pmol/L. STS was continued after dialysis for 4 months ultimately with complete ulcer healing (Figure 2).

## 3. Discussion

We reported on 2 cases of CUA with AGMA from IV STS. Several prior case reports have described AGMA during STS treatment for CUA (Table 1) [2–6] with one leading to cardiac arrest from STS 25 g IV daily in a patient with CKD 4 [6]. Two patients with CKD 4 each received a cumulative dose of 25 g daily [5, 6]. Of ESRD patients, one was on PD and was treated with 25 g IV three times weekly; two patients were on HD and were treated, respectively, with 25 g IV three times weekly and 5 g IV four times weekly. All five cases were female and of the cases that specified race, all four were Caucasian. Although the incidence of STS induced AGMA is not well studied, one cohort demonstrated 4 of 27 (15%) calciphylaxis patients on HD who developed a high AGMA [7]. Dosing varied from 10 g IV three times weekly, 25 g IV three times weekly, and 25 g IV five times weekly. Interestingly, serum pH did not significantly differ between survivors and nonsurvivors. Another study of 172 CUA patients on HD showed a statistically significant rise in serum anion gap without a statistically significant change in serum sodium and bicarbonate levels before, during, and after STS therapy [8].

The means of STS inducing acidemia is unclear though possibly due to the retention of unmeasured sulfate anion and/or formation of thiosulfuric acid in blood [2, 4]. Additionally, an expansion acidosis may also occur due to the large sodium load, since sodium thiosulfate itself is not an acid [5]. Complicating the matter, STS can falsely increase the serum chloride measurement among certain laboratory analyzers, which can mask the presence of a high anion gap [9]. Therefore, serum pH should be monitored during treatment to avoid toxicity.

The mechanism of action of STS in treating CUA is not well established and likely multifactorial. The primary mechanism involves calcium chelation, as STS has been reported to react with calcium to form calcium thiosulfate, which is 250–100,000 times more soluble in aqueous solution than other calcium salts [10]. Additionally, STS demonstrates antioxidant properties that have been shown to improve endothelial dysfunction, promote vasodilation [11], and generate glutathione [3]. Interestingly, the observed acidemia with STS therapy may be an additional mechanism of action, inhibiting the precipitation of calcium and phosphate salts [11].

Thiosulfate has a renal clearance in dogs comparable to creatinine [12]. The half-life of IV thiosulfate in normal dogs was 46.8 minutes and prolonged to 239 minutes in anuric dogs with elevated plasma concentrations detected after at least 16 hours [13]. Lethal doses in animals have ranged from 1 to 4 g/kg and mortality is associated with acidemia, hypernatremia, hypoxia, and hypertension [14, 15].

IV dosing for STS range from 5 to 75 g after HD and is most commonly given at 25 g three times weekly after HD with infusion times between 30 and 60 minutes [16]. Intraperitoneal STS has been described in several reports at doses of 12.5 g–25 g in 2 L dialysate with varying PD intensity [17–19]. One case was complicated by chemical peritonitis [20] but argued that this adverse reaction was due to particulate matter contamination rather than STS [21]. No studies have analyzed the pharmacokinetics of STS with PD and two cases discuss pharmacokinetics with HD [11, 16].

In Case 1, our initial regimen of 25 g IV daily was an aggressive attempt to achieve improved analgesia with cure of the disease. In retrospect, the time STS was administered each day was inconsistent, allowing for potential of overdose. If a relatively high dose or frequency is desired in PD patients, perhaps the acidemia could be remedied by infusing STS IV at the beginning of the nocturnal cycler, increasing exchange volumes, or the patient could be increased to 5-6 manual exchanges over 24 hours on days of administration to increase clearance.

Once again, STS is most commonly administered as 25 g IV three times weekly after HD. However, it is unknown whether a higher dose three times weekly after dialysis or a lower dose given daily is more effective and with less side effects. It seems reasonable to start at a lower dose of 12.5 g IV in HD and PD patients and up to 25 g IV in HD patients three times weekly. Dose titration should be performed through the duration of treatment to achieve a mild AGMA as the induced acidemia can augment the solubility of calcium and possibly aid in ulcer healing. Furthermore, dosing should be adjusted for changes in dialysis modality and intensity to achieve a therapeutic benefit and to avoid toxicity [7]. Currently, there is a scarcity of data regarding intraperitoneal dosing.

In conclusion, STS should be considered in the treatment of CUA. However, little is known about optimal dosing, pharmacokinetics, or adjustments for dialysis patterns and additional studies are required to determine dosing strategies to minimize severe potential side effects.

## Figures and Tables

**Table 1:** Cases of calcific uremic arteriolopathy with an anion gap metabolic acidosis from sodium thiosulfate.

	Patient characteristics	Dose	Duration	Laboratory values	Outcome
Cicone et al. 2004 [2]	69-year-old Caucasian female with ESRD on CAPD for 4 months.	25 g IV 3x/week	8 months	Average HCO_3_ = 15.4 mmol/L Average AG = 26.1 mmol/L	Resolution of pain and reduction in size of subcutaneous plaques.

Selk and Rodby 2011 [3]	65-year old female with ESRD on HD.	25 g IV 3x/week after HD	Not specified	HCO_3_ = 10 mmol/L AG = 44 mmol/L	Acidemia prompted a significant reduction in STS dose. CUA outcome not specified.

Brucculeri et al. 2005 [4]	48-year-old Caucasian female with ESRD on HD.	5 g IV 4x/week after HD	34 months at time of publication.	Average HCO_3_ = 13.1 mmol/L Average AG = 26.7 mmol/L	Excellent wound healing without disease recurrence.

Vanparys et al. 2013 [5]	71-year-old Caucasian female status, after cadaveric renal transplant with CKD 4 secondary to cyclosporine toxicity and chronic allograft dysfunction.	12.5 g IV BID	2 weeks	HCO_3_ = 12 mmol/L AG not specified	STS dose decreased to 7.5 g BID and PO bicarbonate started. Patient showed improved wound healing but died from sepsis.

Mao et al. 2013 [6]	71-year-old Caucasian female with CKD 4.	25 g IV daily	2 weeks	Serum pH <6.80 HCO_3_ = 6 mmol/L AG = 45 mmol/L	Survived 2 cardiac arrests. Patient declined further treatment and was discharged to hospice.

Rein et al. (this report)	49-year-old Caucasian female with ESRD on CAPD.	25 g IV daily	5 months	HCO_3_ = 8 mmol/L, AG = 50 mmol/L	Dose changed to 12.5 mg IV every other day then 12.5 mg weekly. Cured of calciphylaxis in 5 months.
	51-year-old Caucasian male with ESRD started on HD.	25 g IV 3x/week after HD	4 months	HCO_3_ = 18 mmol/L, AG = 27 mmol/L	No change in STS dose. Cured of calciphylaxis in 4 months.
